# Survey on 9 years of anti‐doping controls in horse races in Italy

**DOI:** 10.1111/evj.14496

**Published:** 2025-03-13

**Authors:** Mariana Roccaro, Riccardo Rinnovati, Luca Stucchi, Federica La Rocca, Giuseppe Cascio, Angelo Peli

**Affiliations:** ^1^ Department for Life Quality Studies Alma Mater Studiorum University of Bologna Rimini Italy; ^2^ Department of Veterinary Medical Sciences Alma Mater Studiorum University of Bologna Ozzano dell'Emilia Italy; ^3^ Department of Veterinary Medicine University of Sassari Sassari Italy

**Keywords:** animal welfare, doping, prohibited substance, racehorses

## Abstract

**Background:**

Doping in racehorses is a threat to horse welfare and the integrity of the sport. Despite its relevance, the literature on the prevalence of anti‐doping violations worldwide is limited.

**Objectives:**

To analyse anti‐doping violations in racehorses in Italy.

**Study Design:**

Retrospective observational study.

**Methods:**

Data on horse races, anti‐doping testing, and confirmed violations between 2014 and 2022 were collected and analysed. Positivity rates, most common drug classes and parent drugs, and differences between trotting and galloping horses were investigated.

**Results:**

During the considered 9‐year timeframe, the national laboratory analysed a total of 104,770 samples. A total of 536 horses were confirmed to be positive (92.8% positivity rate at second analysis). The average prevalence over the years was 0.48 ± 0.15% in trotters and 0.50 ± 0.15% in gallopers. Seventy‐seven parent drugs, belonging to 29 different drug classes, were detected. The five most represented drug classes were steroidal anti‐inflammatories (19.0%), stimulants (16.4%), NSAIDs (15.5%), anabolic steroids (9.9%) and sedatives (9.7%). The five most frequent substances were dexamethasone (8.4%), cocaine (7.1%), testosterone (6.5%), caffeine (5.6%) and theophylline (4.1%).

**Main Limitations:**

Our data derive from official analyses performed in compliance with the national regulation on anti‐doping controls in racehorses; information on the concentration of the detected analytes was not available. Testing only the best‐placed horses does not allow for correlating drug administration and improved performance; horses with less chance of winning might slip through the control system with negative consequences on their welfare.

**Conclusions:**

The percentage of confirmed doping violations in horse races in Italy in the 9 years (2014–2022) evaluated in this study was low (0.49 ± 0.15%). Considering the drug classes found most frequently, violations might have been more often the consequence of deliberate administration rather than accidental feed contamination.

## 1 INTRODUCTION

In Italy, all horses taking part in trot and gallop races, managed by the Ministry of Agriculture, Food Sovereignty and Forestry (MASAF), and those officially in training, are eligible for anti‐doping controls. The regulation for the control of prohibited substances, issued in 2002,^1^ has been amended over the years to comply with the international agreements of the International Federation of Horseracing Authorities (IFHA) and the Union Européenne du Trot (UET). Urine and/or blood samples are taken in duplicate (sample A and B) after the race (usually from the first one, three, or five placed horses), from the horses that are injured or die during the race, and, in a small proportion, during training. Before racing, a set of trotting horses (at least 2 or 6, according to the type of race) is also tested to detect the use of alkalinising agents (known as ‘milk shakes’) by measuring total carbon dioxide (TCO_2_) concentration. According to Italian regulations, any substance (its metabolites or isomers) capable at any time of causing an action and/or effect within any mammalian body system is prohibited. A short list of substances is tolerated under defined thresholds and includes only substances endogenous to the horse and substances derived from plants traditionally grazed or harvested as equine feed (Table 1). To avoid inter‐laboratory variability, all the analyses are performed by the MASAF official laboratory, ISO 9001:2015 and ISO 45001:2018 certified, UNI CEI EN ISO/IEC 17025 accredited, on sample A. The list of the testing methods used by the laboratory is published on the ACCREDIA website.^2^ Within 10 days after notification of a positive result, a second analysis is performed on the duplicate sample (sample B) at another accredited laboratory upon request by the horse's trainer or owner.

**TABLE 1:** Complete list of controlled substances and relative thresholds, in urine and plasma, according to the Italian Regulation (Ministry Decree no. 797 of 16 October 2002 and subsequent amendments, in force until 10 September 2023).

Substance	Threshold in urine	Threshold in plasma
Salicylic acid	750 μg/mL	6.5 μg/mL
Arsenic	0.3 μg/mL	‐
Cobalt	0.1 μg/mL	0.025 μg/mL
Dimethyl sulfoxide (DMSO)	15 μg/mL	1 μg/mL
Carbon dioxide (TCO_2_)	‐	36 mmol/L
Hydrocortisone	1 μg/mL	‐
Methoxy tyramine	4 μg/mL	‐
Prednisolone	0.01 μg/mL	‐
Theobromine	2 μg/mL	0.3 μg/mL
Boldenone	0.015 μg/mL (stallions)	‐
Estranediol (nandrolone metabolite)	0.045 μg/mL (stallions)	‐
Testosterone	0.02 μg/mL (geldings) 0.055 μg/mL (fillies, mares)	100 pg/mL (geldings, fillies, mares)

Given the ever‐increasing concern for the welfare of horses and animals in general, doping cases always have great resonance among the public, leading people to fear that doping is a widespread phenomenon in racehorses despite the regulations that are in force. Literature on the prevalence of anti‐doping violations in horses worldwide is scarce. Country‐specific data are available for the UK (1970–1981),^3^ Iran (2002–2003),^4^ Illinois (2004–2009),^5^ Cyprus (2001–2010),^6^ Louisiana (2016–2020),^7^ and the Czech Republic (2010–2019).^8^ Scientific reports on anti‐doping violations in Italy are not available, and we aimed to retrospectively analyse anti‐doping violations reported in racehorses in Italy between 2014 and 2022 and identify prevalence and the substances involved.

## 2 MATERIALS AND METHODS

This is a retrospective observational study examining anti‐doping violations in racehorses in Italy reported between 2014 and 2022. All horses taking part in trotting and galloping races during this timeframe and testing positive for anti‐doping controls were included in the study. Subjects testing negative after second analyses or involved in different disciplines were excluded. Data on the number of races, horses having run, and starts were obtained from an online database for Italian horseracing (HippoWeb.it). The total number of horses tested for prohibited substances was provided by the MASAF. Data on horses that tested positive for anti‐doping controls and the detected substances were retrieved from a publicly available database held by the MASAF.^9^ The detected substances and their metabolites were classified by parent drug and drug class (e.g., benzoylecgonine and ecgonine methyl ester are reported as cocaine—stimulants, oxyphenbutazone as phenylbutazone—NSAIDs, etc.). The detected substances were further categorised as narcotics, anabolic steroids, other zero‐tolerance substances, and other substances tolerated under thresholds. Statistical analysis was performed using Stata BE v.17.0 (StataCorp LLC). In addition to descriptive analyses, differences in the detection of the most common drug classes and parent drugs between trotting and galloping horses were investigated using Pearson's Chi‐squared test or Fisher's exact test (whenever expected frequencies were less than 5). A *p*‐value ≤0.05 was considered significant.

## 3 RESULTS

The number of horses and starts by year and type of race (trot, gallop) is reported in Table 2. The number of trotters was, on average, 1.5 times greater than the gallopers, with an average number of starts per horse 2 times greater for trotters (12 vs. 6).

**TABLE 2:** Number of horses and starts by year and type of race (trot, gallop).

	Year								
	2014	2015	2016	2017	2018	2019	2020	2021	2022
Trot									
No. horses	5,898	5,717	5,655	5,419	5,418	5,395	5,509	5,768	5,774
No. starts	78,451	71,280	74,919	68,913	64,936	62,767	54,693	64,298	62,788
Gallop									
No. horses	3,879	3,768	3,631	3,552	3,697	3,599	3,690	3,764	3,586
No. starts	25,062	25,128	24,017	22,504	22,073	22,466	21,205	23,005	21,697

During the considered 9‐year timeframe, the national laboratory analysed a total of 104,770 samples, collected from 72,922 (69.6%) trotting and 31,848 (30.4%) galloping horses (Table 3). On average, 12.1 ± 0.4% of trotting horses and 15.4 ± 0.8% of galloping horses declared as starting were tested each year. The number of trotting horses tested for TCO_2_ levels before the race was 23,048 (Table 4), that is, representing 3.8 ± 0.3% per year on average.

**TABLE 3:** Number and percentage of horses tested and confirmed positive by year and type of race (trot, gallop).

	Year								
	2014	2015	2016	2017	2018	2019	2020	2021	2022
Trotting									
No. tested (%)	9,279 (11.83%)	9,189 (12.89%)	9,185 (12.26%)	8,340 (12.10%)	7,478 (11.52%)	7,564 (12.05%)	6,811 (12.45%)	7,625 (11.86%)	7,451 (11.87%)
No. confirmed positive (%)	81 (0.87%)	46 (0.50%)	39 (0.42%)	38 (0.46%)	25 (0.33%)	37 (0.49%)	31 (0.46%)	31 (0.41%)	31 (0.42%)
Galloping									
No. tested (%)	3,818 (15.23%)	3,778 (15.04%)	3,644 (15.17%)	3,881 (17.25%)	3,219 (14.58%)	3,464 (15.42%)	3,246 (15.31%)	3,430 (14.91%)	3,368 (15.52%)
No. confirmed positive (%)	22 (0.58%)	16 (0.42%)	18 (0.49%)	21 (0.54%)	20 (0.62%)	7 (0.20%)	21 (0.65%)	21 (0.61%)	12 (0.36%)

**TABLE 4:** Number of trotting horses tested for ‘milk shakes’ and showing increased total carbon dioxide (TCO_2_) concentrations each year.

	Year								
	2014	2015	2016	2017	2018	2019	2020	2021	2022
No. tested (%)	3,022 (3.85%)	3,045 (4.27%)	2,462 (3.29%)	2,623 (3.81%)	2,433 (3.75%)	2,460 (3.92%)	2,015 (3.68%)	2,492 (3.88%)	2,496 (3.98%)
No. confirmed positive (%)	2 (0.07%)	3 (0.10%)	1 (0.04%)	3 (0.11%)	3 (0.12%)	2 (0.08%)	4 (0.20%)	1 (0.04%)	0 (0.00%)

A total of 549 horses (0.4%) tested positive to anti‐doping controls. Second analyses of the B sample were requested in 180 (32.8%) cases; 167 (92.8%) were positive, 9 were negative, and 4 are still ongoing. The total number of positive urine/blood analyses was 536; 71.0% of these positive tests were in trotters, due to the higher number of trotters tested. However, the average prevalence of positive cases between 2014 and 2022 was 0.48 ± 0.15% in trotters and 0.50 ± 0.15% in gallopers (Table 3). Of the trotting horses tested for TCO_2_ levels, 19 (0.08%) showed positivity, ranging from 4 (0.20%) in 2020 to no cases in 2022 (Table 4).

Seventy‐seven parent drugs, belonging to 29 different drug classes, were detected. Of these, 67 (87.0%) were zero‐tolerance substances. Among the substances allowed below the threshold, the most frequently detected were testosterone, cobalt, TCO_2_, Dimethyl Sulfoxide (DMSO), and prednisolone.

Eighteen horses (3.4%) tested positive for more than one drug class; 49 horses (9.1%) tested positive for more than one parent drug. The five most represented drug classes were steroidal anti‐inflammatories (19.0%), stimulants (16.4%), NSAIDs (15.5%), anabolic steroids (9.9%) and sedatives (9.7%) (Table 5). The five most frequent substances were dexamethasone (8.4%), cocaine (7.1%), testosterone (6.5%), caffeine (5.6%) and theophylline (4.1%) (Table 6).

**TABLE 5:** Absolute and relative frequencies of the 15 most prevalent drug classes detected in trotting and galloping horses.

Drug class	Total	Trotting	Galloping	*p*‐Value
	(*n* = 536)	(*n* = 378)	(*n* = 158)	
Steroidal anti‐inflammatories	102 (19.0%)	65 (17.2%)	37 (23.4%)	ns
Stimulants	88 (16.4%)	73 (19.3%)	15 (9.5%)	**
NSAIDs	83 (15.5%)	50 (13.2%)	33 (20.9%)	*
Anabolic steroids	53 (9.9%)	31 (8.2%)	22 (13.9%)	*
Sedatives	52 (9.7%)	35 (9.3%)	17 (10.8%)	ns
Antihaemorrhagics	30 (5.6%)	17 (4.5%)	13 (8.2%)	ns
Erythropoiesis regulators	21 (3.9%)	19 (5.0%)	2 (1.3%)	*
Alkalinising agents	a	19 (5.0%)	a	‐
Anaesthetics	18 (3.4%)	12 (3.2%)	6 (3.8%)	ns
Diuretics	16 (3.0%)	13 (3.4%)	3 (1.9%)	ns
Permeation enhancers	16 (3.0%)	15 (4.0%)	1 (0.6%)	*
Beta‐blockers	9 (1.7%)	9 (2.4%)	0 (0.0%)	ns
Bronchodilators	7 (1.3%)	4 (1.1%)	3 (1.9%)	ns
Opioid analgesics	6 (1.1%)	5 (1.3%)	1 (0.6%)	ns
Muscular relaxants	6 (1.1%)	4 (1.1%)	2 (1.3%)	ns

Abbreviation: ns, not significant.

^a^ Galloping horses are not tested for TCO_2_.

* *p* < 0.05;

** *p* < 0.01.

**TABLE 6:** Absolute and relative frequencies of the 20 most prevalent parent drugs detected in trotting and galloping horses.

Parent drug	Total	Trotting	Galloping	*p*‐Value
	(*n* = 536)	(*n* = 378)	(*n* = 158)	
Dexamethasone	45 (8.4%)	28 (7.4%)	17 (10.8%)	ns
Cocaine	38 (7.1%)	31 (8.2%)	7 (4.4%)	ns
Testosterone	35 (6.5%)	17 (4.5%)	18 (11.4%)	**
Caffeine	30 (5.6%)	25 (6.6%)	5 (3.2%)	ns
Theophylline	22 (4.1%)	18 (4.8%)	4 (2.5%)	ns
Cobalt	21 (3.9%)	19 (5.0%)	2 (1.3%)	*
Phenylbutazone	21 (3.9%)	15 (4.0%)	6 (3.8%)	ns
Triamcinolone acetonide	21 (3.9%)	14 (3.7%)	7 (4.4%)	ns
Betamethasone	20 (3.7%)	15 (4.0%)	5 (3.2%)	ns
Carbon dioxide (TCO_2_)	a	19 (5.0%)	a	‐
Ketoprofen	19 (3.5%)	14 (3.7%)	5 (3.2%)	ns
Acepromazine	18 (3.4%)	10 (2.6%)	8 (5.1%)	ns
Dimethyl sulfoxide (DMSO)	16 (3.0%)	15 (4.0%)	1 (0.6%)	*
Flunixin	16 (3.0%)	9 (2.4%)	7 (4.4%)	ns
Oxazepam	16 (3.0%)	14 (3.7%)	2 (1.3%)	ns
Xylazine	14 (2.6%)	8 (2.1%)	6 (3.8%)	ns
Tranexamic acid	13 (2.4%)	9 (2.4%)	4 (2.5%)	ns
Prednisolone	12 (2.2%)	7 (1.9%)	5 (3.2%)	ns
Furosemide	11 (2.1%)	9 (2.4%)	2 (1.3%)	ns
Stanozolol	10 (1.9%)	7 (1.9%)	3 (1.9%)	ns

Abbreviation: ns, not significant.

^a^ Galloping horses are not tested for TCO_2_.

* *p* < 0.05;

** *p* < 0.01.

In trotters, positivity rates to stimulants (e.g., methylxanthines, cocaine, arsenic) (*p* = 0.005), erythropoiesis regulators (i.e., cobalt) (*p* = 0.04) and permeation enhancers (i.e., DMSO) (*p* = 0.05) were significantly higher, whereas NSAIDs were more frequently detected in gallopers (*p* = 0.03). Overall, 42 (7.8%) horses tested positive for narcotics (i.e., cocaine, morphine) and 53 (9.9%) for anabolic steroids (Table 7). The latter were significantly more frequent in gallopers compared with trotters (*p* = 0.04), particularly testosterone (*p* = 0.003).

**TABLE 7:** Prevalence of positive cases for zero‐tolerance substances and substances with an allowed threshold in trotting and galloping horses.

Type of regulated substance	Total	Trotting	Galloping	*p*‐Value
	(*n* = 536)	(*n* = 378)	(*n* = 158)	
Narcotics	42 (7.8%)	33 (8.7%)	9 (5.7%)	ns
Anabolic steroids	53 (9.9%)	31 (8.2%)	22 (13.9%)	*
Other zero‐tolerance substances	377 (70.3%)	257 (68.0%)	120 (76.0%)	ns
Other allowed‐under‐threshold substances	64 (12.0%)	57 (15.1%)	7 (4.4%)	**

Abbreviation: ns, not significant.

* *p* < 0.05;

** *p* < 0.01.

## 4 DISCUSSION

Anti‐doping controls aim to guarantee the integrity of horseracing and to protect horse welfare.^10^ Several regulations are in place worldwide to prevent and control doping in horse races. The Italian regulation complies with the international agreements of the International Federation of Horseracing Authorities (IFHA) and the Union Européenne du Trot (UET), and it appears to be more restrictive than the guidelines adopted by the Association of Racing Commission International (ARCI), which allow several therapeutic medications under an established threshold (e.g., flunixin, phenylbutazone, ketoprofen, DMSO) and are applied, for example, in some states in the USA and Iran.^4^, ^5^, ^7^ According to the Italian regulation, the list of substances tolerated under a defined threshold is limited to endogenous molecules and substances derived from equine feed.

Under the Italian legal system, horse doping is not only a sports offence but also a criminal offence under Article 544‐ter of the Italian Criminal Code, which punishes anyone who administers narcotics or prohibited substances to animals or subjects them to treatments that are harmful to their health. Several rulings of the Italian Criminal Court of Appeal have stated that ‘the administration of drugs with the ill‐concealed aim of alleviating pain, but in reality to allow a horse to participate in a competition in which it would not have been able to participate in the presence of pain, constitutes animal cruelty because it does not protect the horse's welfare, but rather exposes it to stressful situations and to further risks that may harm its physical and mental state even more significantly’ (Section 3, sentence no. 5235/2016 and 35176/2017).

The scientific literature on doping control violations is scant, and positivity rates vary among countries, ranging from 0.31% to 31.4%.^3^, ^4^, ^5^, ^6^, ^7^, ^8^ This variability can be attributed not only to differences in their current regulations but also to drug availability in the different states, the time frame and context in which the studies were conducted, the testing methods and thresholds used, and, no less important, sample size, thus making the interpretation and comparison of these results inevitably biased.

A 5‐year survey of horseraces in Illinois reported 0.45% positivity of 91,808 analysed samples,^5^ whilst in Louisiana 1.01% of 52,909 post‐race samples were found positive during the years 2016–2020.^7^ A study carried out in Cyprus from 2001 to 2010 reported 161 violations of 94,800 starts. This study defined the number of positive cases as considerably high, although the number of tested samples was not reported.^6^ Recently, 5.4% of the horses competing in the Czech Republic were tested over 10 years, and 2.03% were found positive.^8^ Considering the high number of tested samples (104,770) and the restrictiveness of the Italian regulations in terms of tolerated substances, the prevalence of anti‐doping violations found in our study (0.49 ± 0.15%) can be regarded as low and has shown a decrease in trotting horses compared with the beginning of the studied timeframe. Moreover, the 92.8% positivity rate at the second analysis demonstrates that the applied analytical techniques are highly specific.

Although investigated in a smaller proportion of horses, the use of ‘milk shakes’ does not appear to be a widespread practice in Italy (average positivity rate of 0.08%). Alkalinising agents are administered to increase the blood's natural buffering capacity, thus reducing lactic acid accumulation and delaying fatigue. However, the effectiveness of this potentially harmful practice in the horse has been questioned.^11^, ^12^

A wide variety of substances was detected, most of which were classified as having zero‐tolerance (87.0%), thus suggesting that violations might have been more often the consequence of deliberate administration rather than accidental contamination. In the case of therapeutic medications, adherence to withdrawal times is critical to avoid infractions. According to the regulations, therapeutic medications must be ‘supported by a veterinary prescription, stating the date, the horse's name, its microchip and passport number, the type and amount of medicine administered, its dosage, the start and end date of treatment, diagnosis, prognosis, and the subject's suspension time from racing activity’. The administration of any substance is prohibited on the day of the race.

Positivity to anti‐doping controls results in the disqualification of the horse from the race and a suspension of at least 1 month. Suspension from racing (and breeding) is increased to 2 years in case of positivity to anabolic steroids or growth hormones. The trainer is sanctioned with a temporary suspension of 2–12 months and a fine of €500–6000. The penalties are doubled in case of positivity to anabolic steroids and tripled in case of positivity to narcotics or unauthorised substances. In our study, 95 horses (17.7%) were doped with anabolic steroids or narcotics, thus resulting in harsher punishments. In contrast to other countries, positivity to methocarbamol (*n* = 5), morphine (*n* = 1), and clenbuterol (*n* = 0) was very limited in our sample population.^4^, ^7^, ^8^

Anabolic steroids were reported in three other studies.^3^, ^6^, ^7^ These compounds have long been used in racehorses to enhance muscle growth and athletic performance. Unlike other drug classes, the effects of anabolic steroids can outlast their presence in the body.^13^ In our study, positivity to anabolic steroids, particularly testosterone, was significantly higher in gallopers (13.9%) than in trotters (8.2%). This is likely related to the different types of exercise required, which is mostly anaerobic for gallopers and aerobic for trotters. The former might therefore benefit more from increased muscle mass. Furthermore, testosterone administration has been associated with aggressive behavioural changes, which can be dangerous in harness races.^13^ Although their beneficial effects on human athletes have been widely demonstrated, studies conducted on horses are limited and report conflicting results. Hence, their use in racehorses appears to be rather based on personal opinions and experiences.^14^, ^15^

Cocaine was always detected in the form of its metabolites (benzoylecgonine and ecgonine methyl ester), consistent with its rapid metabolism.^16^ Cocaine has both local anaesthetic and psychostimulatory effects and has been shown to increase the respiratory rate and locomotor activity of horses.^17^, ^18^ However, as already documented in humans,^19^ the detection of cocaine metabolites might also be the result of inadvertent contamination by human drug abusers who handle or feed the horse. Doses as small as 2.5 mg (much lower than the highest no‐effect dose proposed by Queiroz‐Neto et al.^18^) could result in a positive test by GC–MS analysis up to 24 h after administration.^20^

Methylxanthines (caffeine, theophylline and theobromine) were the most detected stimulants.^3^, ^4^, ^6^, ^7^, ^8^ They have broncho‐dilative, vasodilative, and analeptic effects.^21^ Consequently, their significantly higher frequency in trotters may be the result of their role in boosting aerobic metabolism. Being naturally present in some plants, their presence may be either the result of intentional administration or the accidental intake of contaminated feed. For this reason, international thresholds have been established. A recent review argues that the threshold for caffeine is too low; it points out the absence of a threshold for the metabolite paraxanthine and suggests the establishment of a cumulative limit for all methylxanthines to overcome these limitations.^22^

Cobalt is an essential trace element in vertebrates commonly found in nature. Positivity to this substance was significantly higher in trotters, in which it is likely used for its action as an erythropoiesis promoter. However, although the effect of persistently high plasma cobalt levels on red blood cells and exercise performance has been demonstrated in other species, there is no such evidence in horses.^23^

Steroidal and non‐steroidal anti‐inflammatory drugs were among the most frequent violations, and they are usually administered to alleviate inflammation and pain, particularly associated with musculoskeletal disorders. Despite being substances with recognised therapeutic effects, their misuse to mask underlying health issues impacts the horse's safety and welfare.^24^ In other countries, the most frequently reported violations include permitted therapeutic medications,^4^, ^5^, ^7^ thus suggesting the great potential for abuse and highlighting the critical importance of establishing scientifically sound limits of detection and withdrawal times.^10^

Positivity to DMSO can also be explained by its analgesic and anti‐inflammatory properties associated with its permeation‐enhancing effect.^25^, ^26^ Although approved in veterinary medicine for topical administration only, over‐threshold levels in urine/plasma that were significantly higher in trotters may be explained by other routes of administration, such as intravenous infusion, aiming to obtain a greater analgesic/anti‐inflammatory effect.

Italian regulations were recently updated in 2023, with a new classification of substances.^27^ Category A includes a group of substances whose administration is prohibited throughout the horse's life (unauthorised substances, anabolic agents, peptide hormones, growth factors and related substances, hormones and metabolic modulators) and substances endogenous to the horse that are tolerated under a defined threshold (testosterone, estranediol, boldenone). Category B comprises a wide range of substances whose presence in the horse is prohibited under any circumstances on the day of the race, and during training (in the latter case, unless justified by a veterinary prescription). Substances derived from plants traditionally grazed or harvested as equine feed fall under this category and are tolerated below threshold; however, DMSO and theobromine are no longer included in this list. An additional change is that, besides testing the best‐placed horses after the race, the competent authority may order pre‐race random tests.

In conclusion, the percentage of confirmed doping violations in horseraces in Italy in the 9 years (2014–2022) evaluated in this study was low (0.49 ± 0.15%). However, testing only the best‐placed horses does not allow for correlating drug administration and improved performance; although a doped horse might have a higher chance of winning, horses with lower chances of achieving good results might also be doped, so their participation is equally fraudulent and detrimental to their welfare. The implementation of random checks before the race and during training might help overcome these limitations.

This is the first study on anti‐doping violations in Italy. Besides its national relevance, this study is of global interest as it establishes a benchmark to inform researchers and stakeholders in other countries. Description of the most commonly detected substances may be useful to the racing community in identifying the drugs most prone to abuse. An improved understanding of anti‐doping regulations might facilitate the work of trainers and veterinarians and improve compliance (i.e., passively following the prescribed rules); however, working towards adherence (i.e., actively choosing to follow the prescribed rules) and behaviour change represent a key element in achieving better end results.

## FUNDING INFORMATION

Not applicable.

## CONFLICT OF INTEREST STATEMENT

The authors declare no conflicts of interest.

## AUTHOR CONTRIBUTIONS

**Mariana Roccaro:** Writing – original draft; writing – review and editing; data curation; formal analysis; visualization; methodology. **Riccardo Rinnovati:** Conceptualization; methodology; writing – review and editing; supervision. **Luca Stucchi:** Writing – original draft; writing – review and editing; formal analysis. **Federica La Rocca:** Investigation; data curation; writing – review and editing. **Giuseppe Cascio:** Writing – review and editing; supervision. **Angelo Peli:** Conceptualization; methodology; writing – review and editing; supervision; resources.

## DATA INTEGRITY STATEMENT

Mariana Roccaro had full access to all the data in the study and takes responsibility for the integrity of the data and the accuracy of the data analysis.

## ETHICAL ANIMAL RESEARCH

Research ethics committee oversight is not required by this journal: The data used are publicly available.

## INFORMED CONSENT

Not applicable.

## Data Availability

Part of the data that supports the findings of this study is openly available from the Ministry of Agriculture, Food Sovereignty and Forestry (MASAF) at https://www.politicheagricole.it/flex/cm/pages/ServeBLOB.php/L/IT/IDPagina/21845. Restrictions may apply to the data provided by HippoWeb.it and MASAF.
